# Numbers, characteristics, and medical complexity of children with life-limiting conditions reaching age of transition to adult care in England: a repeated cross-sectional study [version 1; peer review: 2 approved]

**DOI:** 10.3310/nihropenres.13265.1

**Published:** 2022-04-08

**Authors:** Stuart Jarvi, Gerry Richardson, Kate Flemming, Lorna K Fraser

**Affiliations:** 1 Martin House Research Centre, University of York, York, YO10 5DD, UK; 2 Centre for Health Economics, University of York, York, YO10 5DD, UK; 3 Department of Health Sciences, University of York, York, YO10 5DD, UK

**Keywords:** Life-limiting conditions, Transition to adult care, Medical complexity, Healthcare use, Palliative care

## Abstract

**Background:**

The number of children with life-limiting conditions in England is known to be increasing, which has been attributed in part to increased survival times. Consequently, more of these young people will reach ages at which they start transitioning to adult healthcare (14-19 years). However, no research exists that quantifies the number of young people with life-limiting conditions in England reaching transition ages or their medical complexity, both essential data for good service planning.

**Methods:**

National hospital data in England (Hospital Episode Statistics) from NHS Digital were used to identify the number of young people aged 14-19 years from 2012/13 to 2018/19 with life-limiting conditions diagnosed in childhood. The data were assessed for indicators of medical complexity: number of conditions, number of main specialties of consultants involved, number of hospital admissions and Accident & Emergency Department visits, length of stay, bed days and technology dependence (gastrostomies, tracheostomies). Overlap between measures of complexity was assessed.

**Results:**

The number of young people with life-limiting conditions has increased rapidly over the study period, from 20363 in 2012/13 to 34307 in 2018/19. There was evidence for increased complexity regarding the number of conditions and number of distinct main specialties of consultants involved in care, but limited evidence of increases in average healthcare use per person or increased technology dependence. The increasing size of the group meant that healthcare use increased overall. There was limited overlap between measures of medical complexity.

**Conclusions:**

The number of young people with life-limiting conditions reaching ages at which transition to adult healthcare should take place is increasing rapidly. Healthcare providers will need to allocate resources to deal with increasing healthcare demands and greater complexity. The transition to adult healthcare must be managed well to limit impacts on healthcare resource use and improve experiences for young people and their families.

## Introduction

The number of children with life-limiting conditions in England has increased over the past two decades^1,2^. These conditions often involve medical complexity and include conditions that inevitably lead to premature death and also life-threatening conditions that may result in premature death, but may also be cured (e.g. cancer)^3^.

These increases in prevalence of life-limiting conditions have been attributed, at least in part, to increased survival^1,2,4,5^. A consequence of this is the expectation that more children with life-limiting conditions will survive long enough to transition from paediatric to adult healthcare, something that typically happens from 14 to 19 years in the UK^6–8^. The transition - and associated problems - have been an area of increasing research and policy interest, with variations in experience identified between conditions and availability and remit of local services^7,9–13^. Providing good transition care is important for efficient use of health services and reducing emotional trauma for young people and their families^14–19^.

Concepts of medical complexity, indicative of the need for ‘extra time, expertise, and resources necessary to achieve optimal health outcomes’^20^, have been used as a tool to identify young people, with life-limiting or other chronic conditions, who have extensive healthcare needs^20–23^. These children have been shown to be major users of healthcare across multiple specialties^24,25^ and the related group of children with disabilities has been shown to also have complex care needs and healthcare use correlating with complexity^23^.

High healthcare use across multiple specialties associated with medical complexity, coupled with the acknowledged challenges in transitioning children with life-limiting conditions into adult care, presents challenges for service providers. There is a need, for good service planning, for knowledge on how many young people with life-limiting conditions are at ages where transition to adult care should take place, whether their characteristics (such as category of health condition or ethnic group) are changing over time and how complex their medical needs are.

While previous studies have estimated the numbers of young people with life-limiting conditions, including within age groups close to transition ages^1,2^, these studies have not differentiated between those with conditions diagnosed in childhood (and therefore likely to undergo transition) and those with conditions diagnosed in late adolescence (who may go directly into adult care). There are no studies assessing the medical complexity of this population on a national basis in England.

This study uses routinely collected hospital records to assess national trends in the numbers, characteristics, and medical-complexity of young people with life-limiting conditions reaching the age to transition to adult healthcare in England.

## Methods

### Ethical approval

Health Research Authority ethical approval was obtained for this study from Wales Research Ethics Committee 5 (REC reference 20/WA/0149, chair Dr Jason Donal Walker, Integrated Research Application System project ID 282131).

### Patient and public involvement

The Martin House Research Centre Family Advisory Board^26^, which comprises parents and carers who either have or had children with life-limiting conditions, was consulted about the challenges of transition and the complexity of healthcare in this population. Their input helped to determine the aspects of healthcare assessed in this study.

### Data

Hospital Episode Statistics (HES) (records of hospital care in England funded by the National Health Service (NHS)^27^) data were requested from NHS Digital. Inpatient (1 April 2006 - 31 March 2019), outpatient (1 April 2006 - 31 March 2019) and Accident & Emergency (A&E, 1 April 2007 - 31 March 2019) were requested for all children and young people aged 12–23 years at any point between 1 April 2007 - 31 March 2019.

### Data management

Data were managed in Microsoft SQL Server 2019. Other SQL servers such as MariaDB (MariaDB, RRID:SCR_021763) or MySQL can also be used. Analyses and graphs were produced using R project version 3.5.3 (R Project for Statistical Computing, RRID:SCR_001905).

#### Population of interest

This was a repeated cross-sectional study. In each year, individuals were included if they met the following criteria:

- Had a diagnosis of a life-limiting condition in HES inpatient or outpatient records, matching a previously developed1 International Classification of Diseases, 10th Edition^28^ (ICD-10) coding framework in that year or a previous year while aged 16 years or younger. Perinatal diagnoses from the framework were excluded as, without subsequent life-limiting diagnoses in another category, they were not deemed indicative of an ongoing life-limiting condition at transition ages (Table 1).
- Had a HES record in the year while aged 14–19 years and was a resident in England.

Individuals were excluded in a year if they:

- Had only a non-central nervous system cancer life-limiting condition diagnosis (see Table 1) and were first diagnosed more than five years earlier (those having another life-limiting condition diagnosis were not excluded). The rationale for this was that few young people with a non-central nervous system cancer diagnosis more than five years earlier would still be considered life-limited^1^.

#### Demographic data

Age for each person in each year was set to the age of the first record (inpatient, outpatient, or A&E) for that person in each year. Sex was assigned as the most commonly recorded sex in the data. Ethnic group was recorded in the data based on 2001 census groups^29^. These were collapsed to eight groups (White, Indian, Bangladeshi, Pakistani, Black, Chinese, Mixed and Other) to avoid small numbers and then each person was assigned to the most commonly recorded group. Government Office Region of residence was assigned according to the first non-missing value recorded in each year. In the event of no non-missing values in a year, the value from the nearest previous year with a non-missing value was used. Deprivation category was assigned based on the first non-missing Lower Super Output Area (LSOA - a geographic area of, on average, 1500 residents, although sizes vary) of residence recorded in each year - deprivation categories were assigned by population weighting so that approximately 20% of 14–19-year-olds in the general population were in each group. In the event of no non-missing LSOA values in a year, the value from the nearest previous year with a non-missing value was used.

#### Study period

**Figure 1:**
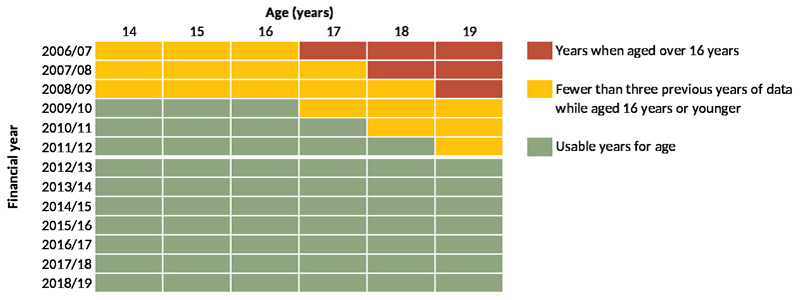
Years of data that could be used in the analysis. Years of available data showing the years in which, for each age in that year, there were data for at least three earlier years while a young person of that age was aged 16 years or younger.

Data from 1 April 2006 to 31 March 2019 were used to determine inclusion eligibility (i.e., analysed for presence of life-limiting conditions and other diagnoses). However, only data from 1 April 2012 to 31 March 2019 are presented, due to left-edge effects, ensuring that all included individuals had at least three years of data aged 16 years or younger in which a life-limiting condition - and eligibility for inclusion - could be detected (Figure 1).

#### Numbers and characteristics of young people of transition age

Numbers of young people aged 14–19 years and known to be present in England (i.e., with an inpatient, outpatient, or A&E record while a resident in England) were calculated each year, overall and by age, sex, ethnic group, Government Office Region of residence and deprivation category.

#### Medical complexity

**Figure 2:**
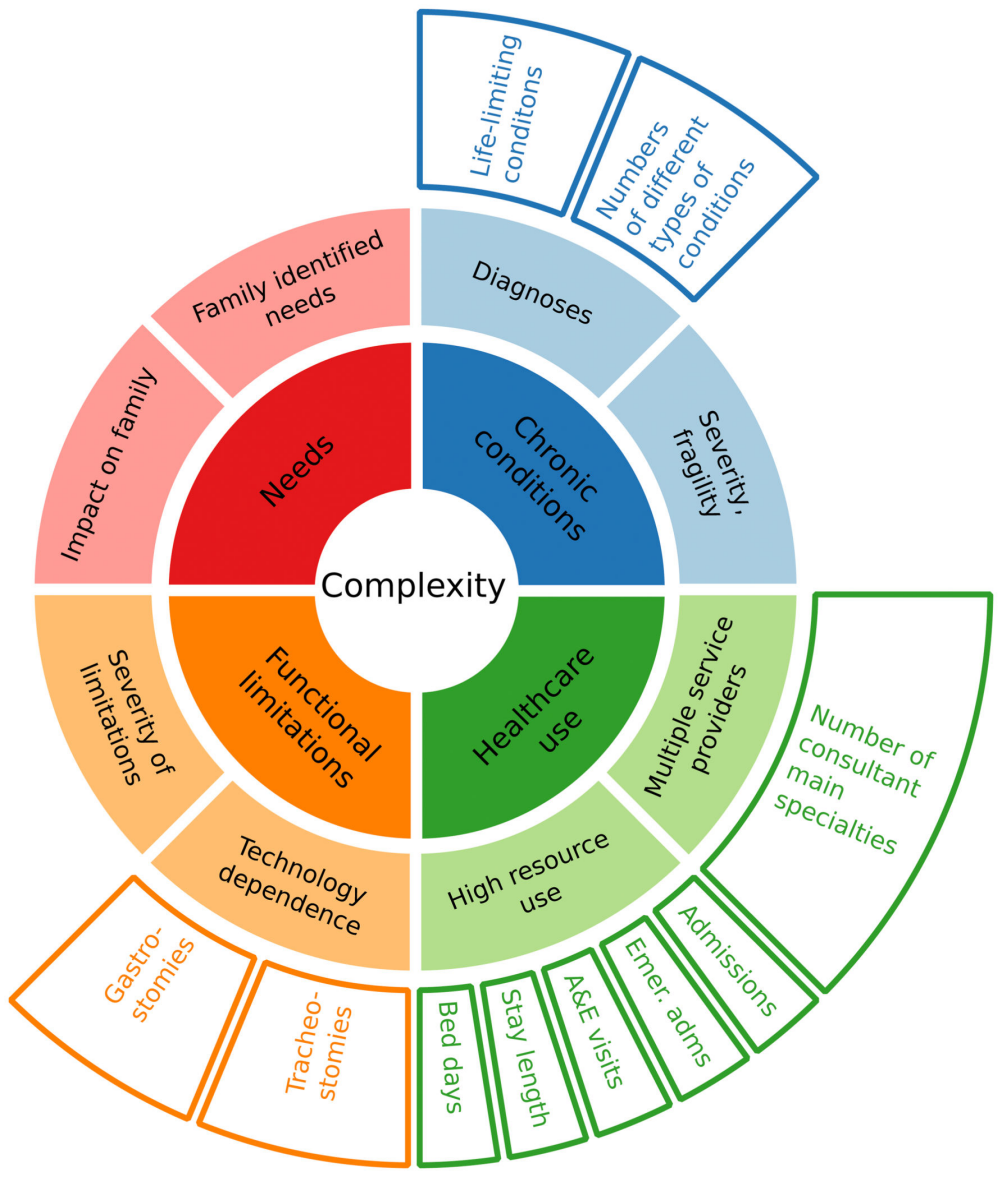
Conceptualisation of complexity and relevant data. Inner two rings, adapted from earlier work^20^, show conceptualisation of complexity. Outer ring shows relevant measures in the data.

Analyses of medical complexity drew on earlier work^20^, matching concepts of complexity to the available data (Figure 2). While the data lack information on family identified needs or impacts on the family, the HES data do provide insights on the other three main concepts of presence of chronic conditions, healthcare use and functional limitations. These were measured as follows.

##### Chronic conditions

This aspect of complexity was assessed through the presence of life-limiting conditions, as described above, and also a measure of the number of distinct categories of conditions (including life-limiting and other chronic conditions) using previously developed^30^ groupings for chronic conditions (Table 2 - explicitly perinatal diagnoses were again excluded as they were not relevant in a population aged 14–19 years). For distinct categories of chronic conditions, in each year for each person the total number of diagnostic categories recorded in inpatient and outpatient records in that, or previous years, was calculated.

##### Healthcare use - multiple service providers

The ‘multiple service providers’ aspect of complexity was assessed through the number of distinct consultant main specialties (MAINSPEF field in inpatient and outpatient datasets, as detailed in the Hospital Episode Statistics Technical Output Specification) recorded for each person in each year in the inpatient and outpatient data. Similar paediatric and adult specialties were considered a single specialty (Table 3) to prevent any variations in the numbers in paediatric or adult care from skewing results; all other unique specialty codes were considered distinct.

##### Healthcare use - high resource use

The ‘high resource use’ aspect of complexity was assessed through different measures of hospital events: total A&E visits, inpatient admissions, emergency admissions and inpatient bed days for each person in each year. Length of stay was also assessed in each year.

##### Functional limitations

Technology dependence was assessed through the numbers of young people with life-limiting conditions with a gastrostomy or tracheostomy present in each year. Insertion (permanent or temporary), attention to or removal of a gastrostomy or tracheostomy was considered evidence of presence in a given year. Permanent insertions were assumed to remain in later years until there was evidence of removal. Presence, insertion and removal of gastrostomies and tracheostomies were identified through ICD-10 diagnostic codes^28^ and OPCS Classification of Interventions and Procedures Version 4 (OPCS-4) procedure codes recorded in the inpatient and outpatient data (Table 4).

##### Overlaps between measures of complexity

Finally, the interconnectedness of measures of complexity was assessed by using UpSet graphs, using the R package, UpSetR version 1.4.0. UpSet graphs are an alternative to Venn or Euler diagrams, which show sizes of intersections between many different sets^31^. Set sizes are shown with bar graphs to the left of set names, while a matrix shows combinations of intersections and bar graphs above the matrix show the size of each intersection. To reduce the number of comparison groups for simplicity in the UpSet graph matrix, analyses were limited to indications of high complexity: intersections between membership of approximately the top 10% of each measure in the final year (2018/19) were compared to see whether being in the top 10% on one measure was indicative of being in the top 10% for another. In addition, it was intended to use only one measure for each of the four second level aspects of complexity for which data were available: diagnoses, multiple service providers, high resource use and technology dependence (middle ring, Figure 2). Where an aspect of complexity had multiple measures (e.g., technology dependence, high resource use) UpSet graphs were used to analyse overlap between individual measures.

## Results

### Numbers and characteristics of young people aged 14–19 years with life-limiting conditions

**Figure 3:**
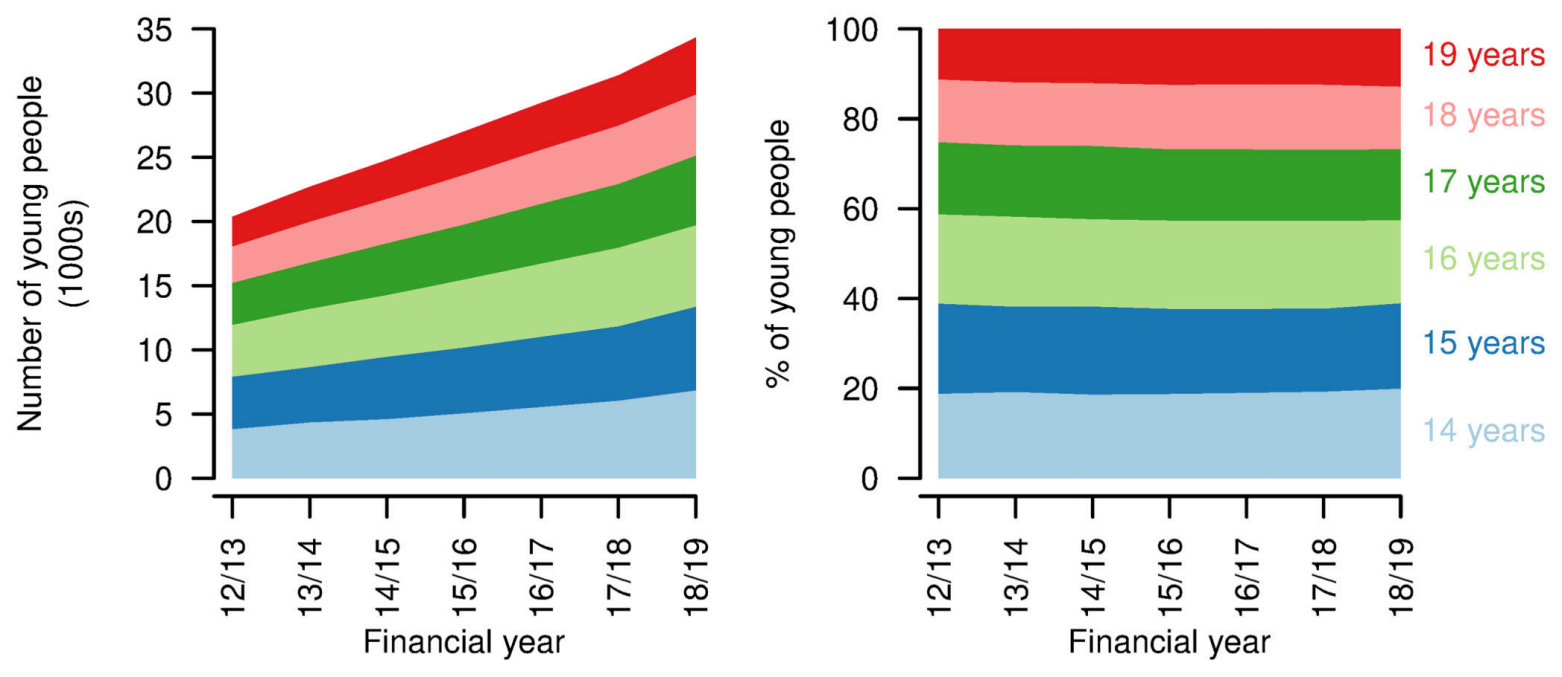
Numbers and ages of young people aged 14–19 years with life limiting conditions. Numbers (left) and proportions (right) of young people aged 14–19 years with a life-limiting condition, by age, by year.

A total of 121626 young people with life-limiting conditions diagnosed while aged 16 years or younger were identified in the data. From financial year 2012/13 to 2018/19, the number of young people aged 14-19 years with a life-limiting condition diagnosed at age 16 years or younger increased from 20363 to 34307 (Figure 3). Proportions in each year of age remained similar over time.

**Figure 4:**
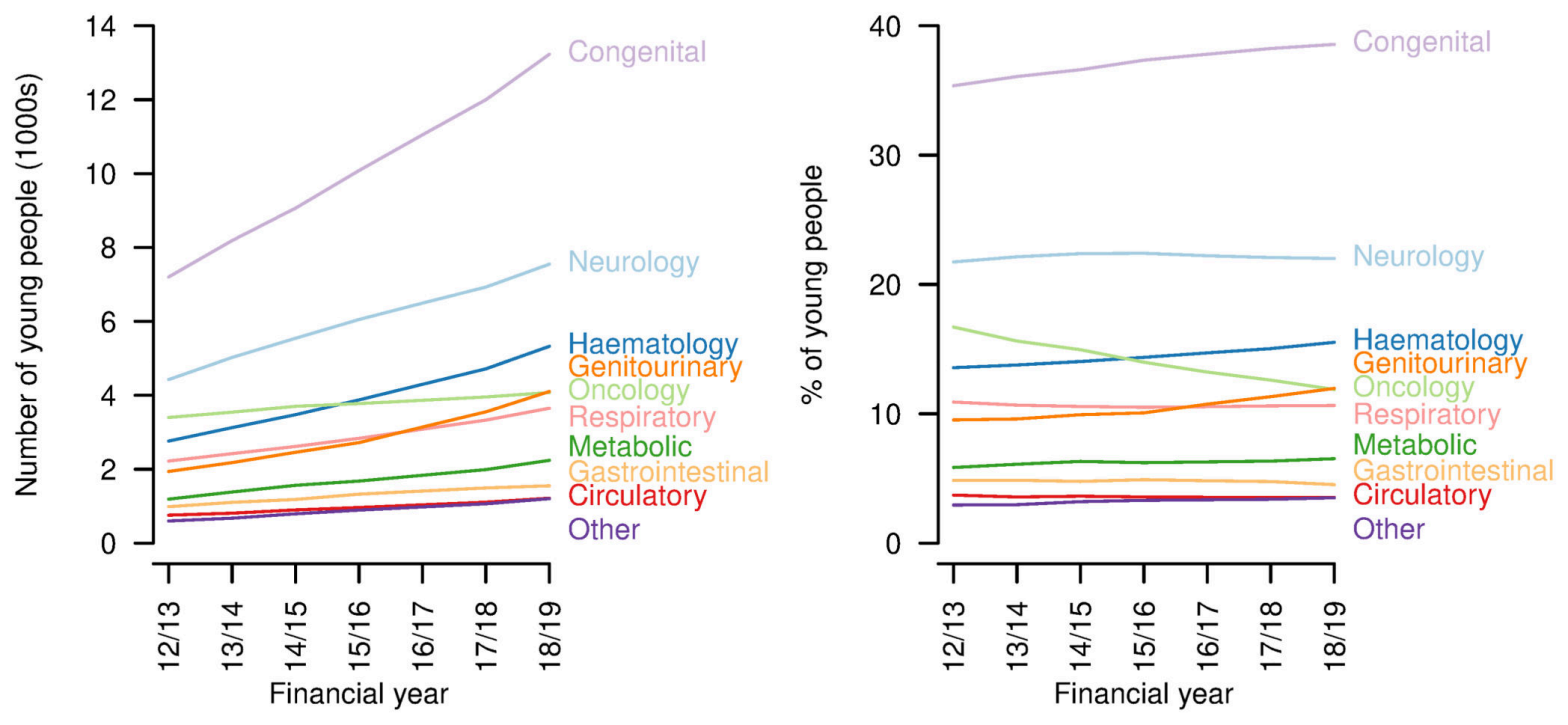
Categories of conditions of young people aged 14–19 years with life limiting conditions. Numbers (left) and proportions (right) of young people aged 14–19 years with a life-limiting condition with each category of condition, by year.

There were increases over the study period in the number of young people aged 14-19 years with each of the categories of life-limiting conditions (Figure 4). Congenital conditions remained the largest group throughout, increasing from 7201 in 2012/13 to 13230 in 2018/19. The proportion with congenital (2012/13: 35.4%; 2018/19: 38.6%), haematology (2012/13: 13.6%; 2018/19: 15.5%) and genitourinary conditions (2012/13: 9.5%; 2018/19: 12.0%) increased and the proportion with oncology conditions - for which a five-year limit from first diagnosis was imposed, except for central nervous system tumours - decreased (2012/13: 16.7%; 2018/19: 11.9%) despite an increase in absolute numbers (2012/13: 3401; 2018/19: 4077). The proportions with other categories of condition remained largely constant.

**Figure 5:**
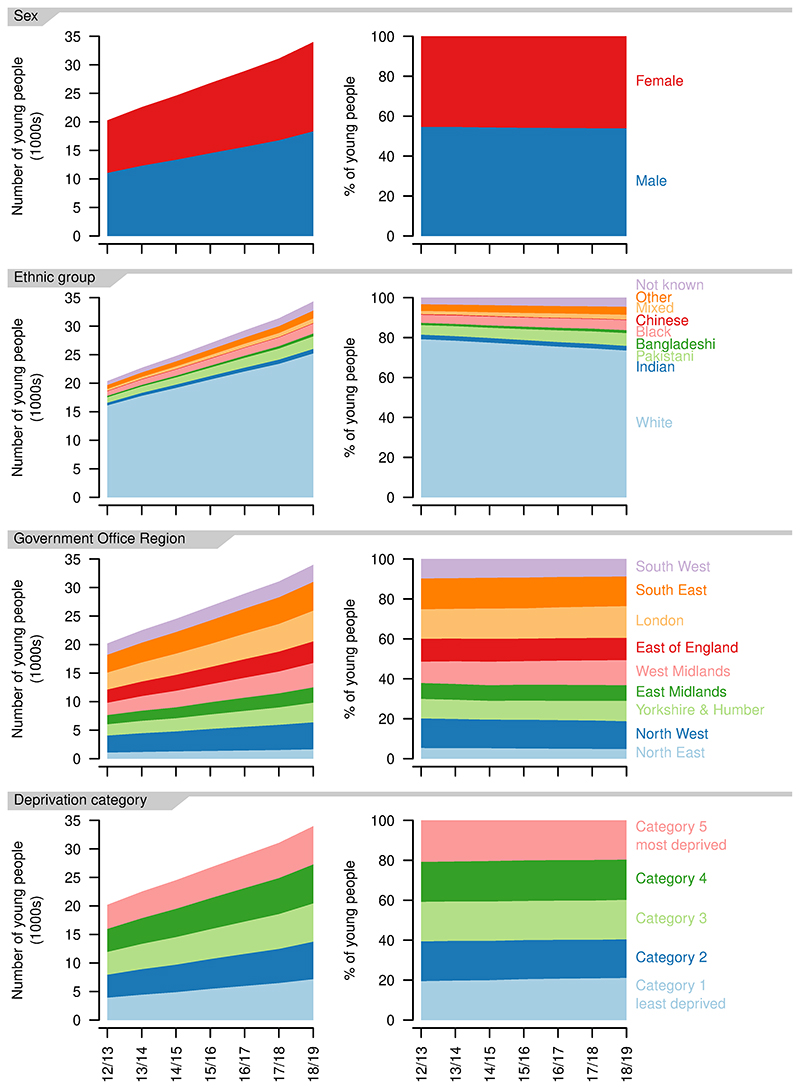
Demographics of young people aged 14–19 years with a life-limiting condition. Numbers (left) and proportions (right) for each recorded sex, ethnic group, Government Office Region of residence and deprivation category, by year.

The balance between recorded sexes remained similar over the study period, with more male than female patients (2012/13: 54.6% versus 45.4%; 2018/19: 54.0% versus 46.0%, Figure 5).

The number of young people in each ethnic group increased over the study period, but the proportion in the White ethnic group decreased (2012/13: 79.2%; 2018/19: 73.6%) as did the proportion in the Chinese ethnic group (2012/13: 0.31%; 2018/19: 0.22%, Figure 5). All other ethnic groups increased as a proportion, with the largest increases in the Mixed (2012/13: 1.6%; 2018/19: 2.4%) and Pakistani ethnic groups (2012/13: 4.8%; 2018/19: 6.4%).

There were increases in the number of young people aged 14-19 years with life-limiting conditions in each of the Government Office Regions (Figure 5). The largest proportional increase was in the West Midlands, where numbers grew by 98% over the study period (2012/13: 2319; 2018/19: 3785) and the smallest in the South West, where numbers grew by 51% (2012/13: 1957; 2018/19: 2959)

There were small variations in the proportion of young people in each deprivation category (Figure 5), with small movements towards less deprived categories, e.g., increases in those in group one (2012/13: 19.5%; 2018/19: 21.1%) and decreases in those in group five (2012/13: 20.8%; 2018/19: 19.6%). Overall, there was a very even distribution between deprivation categories.

### Medical complexity

#### Chronic conditions

**Figure 6:**
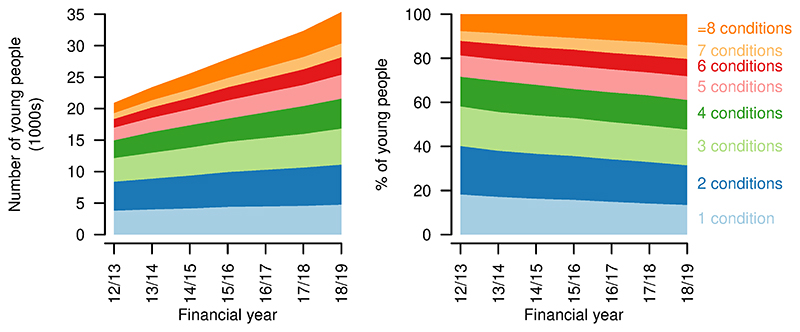
Numbers of chronic conditions for young people aged 14–19 years with a life-limiting condition. Numbers (left) and proportions (right) of young people aged 14–19 years with a life-limiting condition having different numbers of chronic condition categories recorded, by year.

The number of distinct types of chronic condition (including life-limiting conditions) increased over the study period, with the largest proportional increase in the number of young people having eight or more chronic condition categories recorded, up from 7.6% in 2012/13 to 14.0% in 2018/19 (Figure 6). There was a decrease in those with only one chronic condition category recorded, from 18.2% in 2012/13 to 13.5% in 2018/19.

#### Healthcare use - multiple service providers

**Figure 7:**
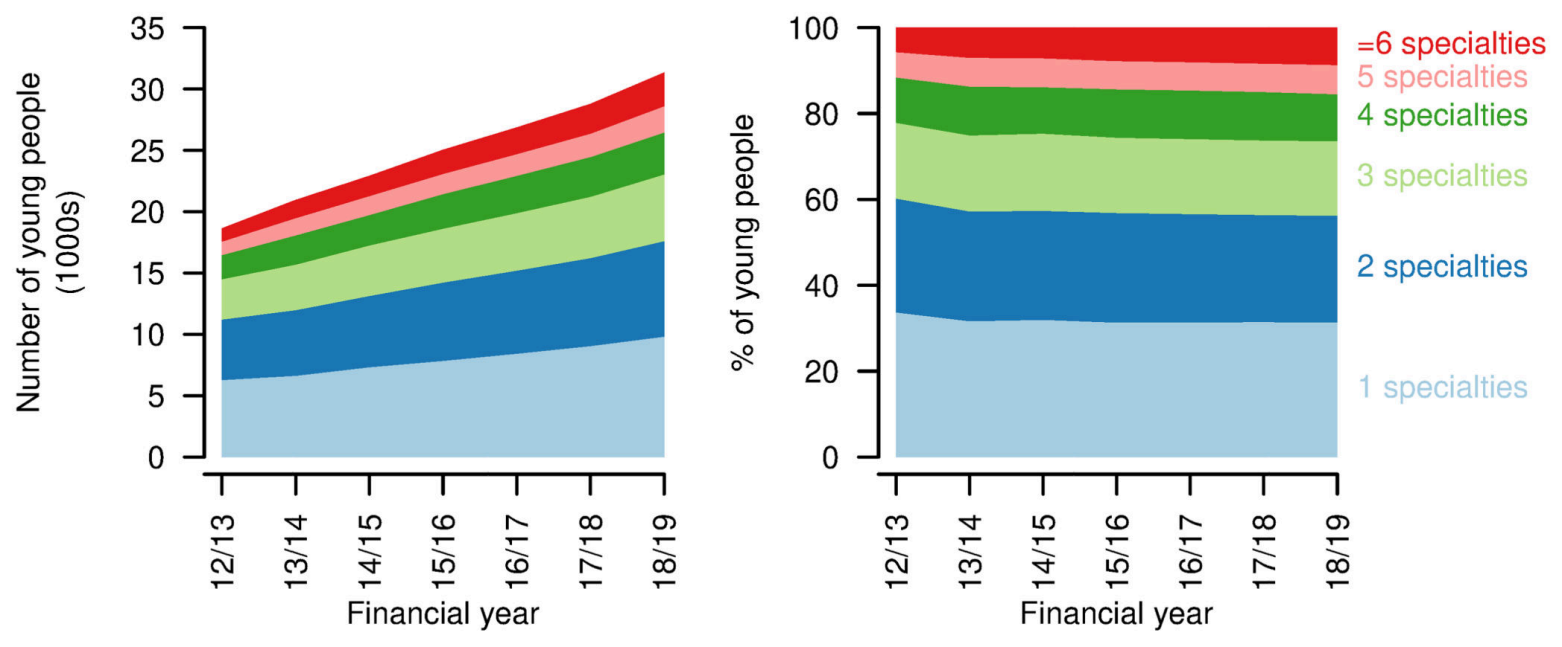
Numbers of main consultant specialties for young people aged 14–19 years with a life-limiting condition. Numbers (left) and proportions (right) of young people aged 14–19 years with a life-limiting condition receiving treatment from consultants under different numbers of consultant main specialties in each year.

An increased proportion of young people with life-limiting conditions were treated by consultants across six or more main consultant specialties (2012/13: 7.9%; 2018/19: 11.0%, Figure 7). There was a fall in the proportion treated by consultants with four or fewer main consultant specialties and a small increase in those treated by consultants with five consultant specialties (2012/13: 7.4%; 2018/19: 7.9%).

#### Healthcare use - high resource use

**Figure 8:**
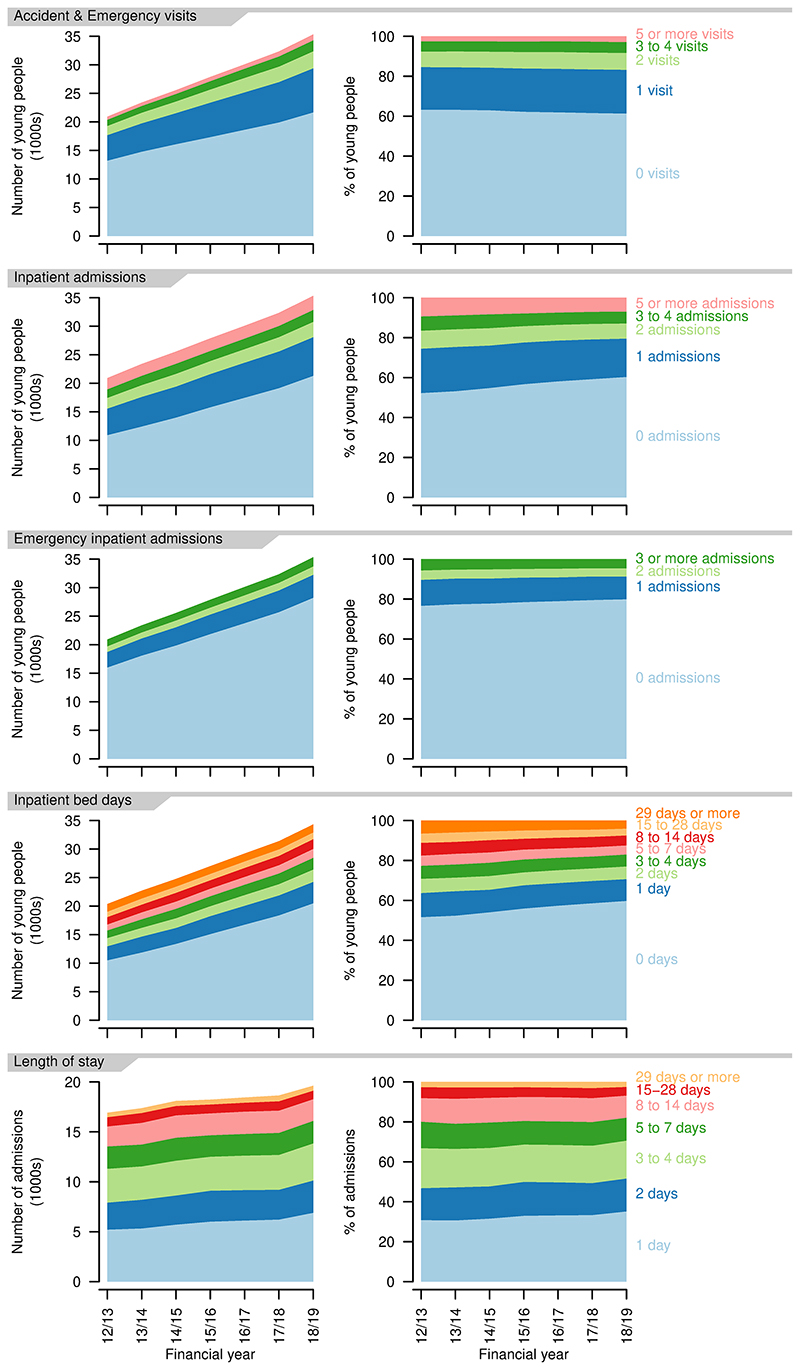
Indicators of high resource use for young people aged 14–19 years with a life-limiting condition. First four rows: numbers (left) and proportions (right) of young people having different numbers of Accident & Emergency visits, inpatient admissions, emergency inpatient admissions, bed days in each year. Final row: numbers (left) and proportions (right) of inpatient admissions of differing lengths in each year.

There was a proportional (and absolute) increase in A&E visits per person per year, with a small drop in those having no A&E visits in a year (2012/13: 63.2%; 2018/19: 61.4%, Figure 8). Total A&E visits increased from 15241 in 2012/13 to 28019 in 2018/19.

Inpatient admissions per person per year decreased over the study period, with increases in those having no admissions in a year (2012/13: 52.8%; 2018/19: 60.3%, Figure 8). Proportions with more admissions reduced correspondingly. There was also a decrease in emergency admissions per person, although to a lesser extent, with an increase in those with no emergency admissions (2012/13: 76.6%; 2018/19: 79.9%, Figure 8). The proportion of young people with no inpatient bed days in a year increased over the study period (2012/13: 51.6%; 2018/19: 59.8%). The group with 29 or more inpatient bed days decreased proportionally the most (2012/13: 6.5%; 2018/19: 4.0%). Total bed days increased from 142557 in 2012/13 to 157298 in 2018/19.

Length of stay (for those spending at least one night in hospital - i.e., excluding day cases) also decreased slightly, with more young people having single night stays (2012/13: 20.8%; 2018/19 35.2%, Figure 8). There was, however, also an increase in the longest stays, of 29 days or more, up to 2017/18, at least (2012/13: 2.6%; 2017/18: 3.1%). Day cases increased from 62.3% to 65.0% of admissions over the same period.

#### Functional limitations

Numbers of young people with gastrostomies or tracheostomies increased over the study period (gastrostomies: 2012/23: 1801, 2018/19: 3143; tracheostomies: 2012/23: 208, 2018/19: 357; Figure 9). However, the proportions changed little for gastrostomies (2012/13: 8.8%; 2018/19: 9.2%, but with variation in both directions over the period) and did not vary between the start and end of the study period for tracheostomies (1.0%).

#### Overlaps between measures of complexity

**Figure 10:**
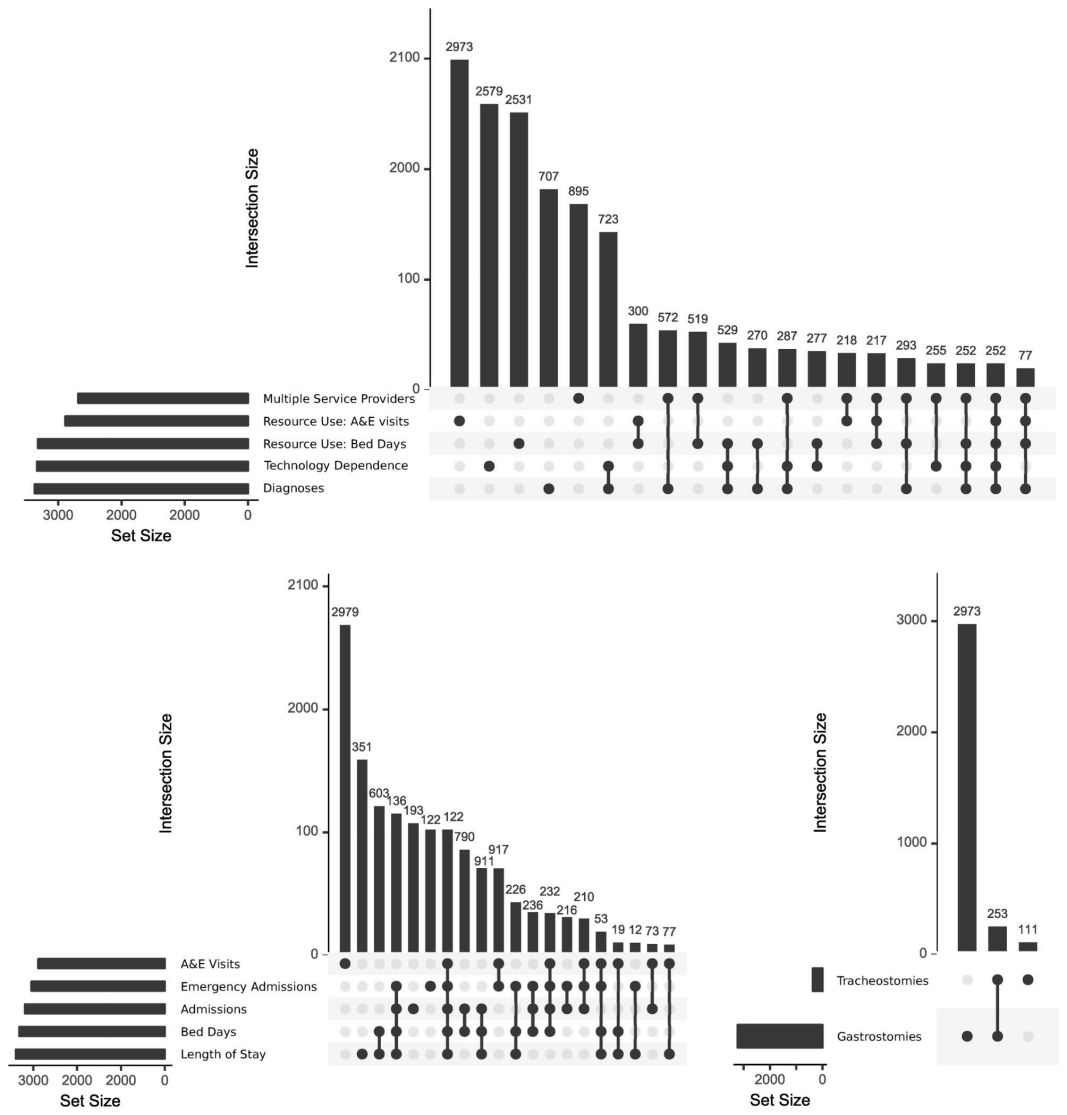
UpSet graphs showing relations between measures of complexity. Intersections between (top) those in approximately the top 10% for each of five indicators of complexity; (bottom left) those in approximately the top 10% in each of the five indicators of high resource use; (bottom right) those with technology dependence. For simplicity, only the largest 20 intersections are shown.

Analysis of intersections between the five measures of high resource use showed little overlap between being in the top 10% for A&E visits and the other indicators (Figure 10) so two indicators were retained - being in the top 10% for A&E visits and being in the top 10% for bed days (this being the most interconnected of the remaining measures and combining aspects of numbers of admissions and length of stay). Presence of a gastrostomy was retained as the measure of technology dependence, due to the low number (approximately 1%) with tracheostomies and the high overlap of those with tracheostomies also having gastrostomies (70%, Figure 10). As a result, membership of the top 10% of five measures in 2018/19 was compared: diagnoses (number of chronic conditions); multiple service providers (number of distinct consultant main specialties); technology dependence (presence of a gastrostomy); resource use (A&E visits and bed days, Figure 10).

The number of young people present in the top 10% of any of the five measures in 2018/19 was 11488 (33% of all young people present in 2018/19). There was limited intersection between the five groups. The five largest categories were for each of the groups alone and totalled 5945 - i.e., 52% of those in the top 10% for any of the five measures were only in the top 10% for one of the measures. Only 1.2% were in the top 10% for all five measures. However, overlap was seen between technology dependence and number of diagnoses - 5% of those in the top 10% on any measure were in the top 10% for both technology dependence and number of diagnoses - and to a lesser extent between bed days and multiple service providers and between diagnoses and multiple service providers. Overall, high numbers of diagnoses (being in the top 10%) was more indicative of being in the top 10% on at least one other measure than the other four measures - 75% of those in the top 10% for number of diagnoses were also in the top 10% for one of the other measures. This compared to 50% for A&E visits, 40% for technology dependence and 35% for bed days.

## Discussion

This study shows increasing numbers of young people with life-limiting conditions reaching the ages at which they transition to adult healthcare. There is also evidence of increased complexity with regard to numbers of recorded chronic conditions and consultants of different specialties seen, but limited evidence for increased instability or hospital healthcare use. It was found that 33% of young people with life-limiting conditions reaching the ages at which they transition to adult healthcare were in the top 10% on at least one of the five measures of complexity, indicating that medical complexity is common in this population.

The increase in numbers of young people with life-limiting conditions at transition ages and with their conditions first diagnosed in childhood is large - 68% larger in 2018/19 than 2012/13. It shows increasing proportions with congenital, haematology and genitourinary diagnoses and a falling proportion with oncology diagnoses who are within five years of first diagnosis (for non-central nervous system tumours) although this group still increased in absolute terms. There has been an increase in those from non-White ethnic groups, particularly the Mixed and Pakistani ethnic groups. By region, the biggest proportional increases have occurred in the West Midlands.

This study is, to the best of the authors’ knowledge, the first attempt to describe medical complexity in young people with life-limiting conditions on a national scale using routinely collected data. It found that there was an increase in the numbers of young people with more different categories of chronic condition recorded, particularly among those with eight or more categories recorded. Similarly, there was an increase in the proportion treated by consultants with six or more distinct main consultant specialties in a year. There was however also an increase in the proportions with no inpatient and no emergency inpatient admissions, although a small increase in those with one or more A&E visits in a year. For length of stay, there were both increases in those having single night inpatient stays and those having the longest stays (29 nights or more). There was an increase in those having no inpatient bed days in the year and a marked decrease in those having 29 or more bed days. The proportion with gastrostomies increased from 8.8% to 9.2%, but there was no notable change in the proportion with tracheostomies.

The lack of overlap between young people in the top 10% of most of the measures of complexity suggests that complexity is, as previously suggested^20,23^, a multi-faceted phenomenon and that multiple measures are required for assessment. Numbers of distinct chronic diagnoses were the strongest indicator of complexity in other categories, with 75% of those in the top 10% for number of diagnoses also in the top 10% for at least one of the other measures.

### Comparisons with previous studies

In common with a previous study covering a similar time period^1^, this study found an increasing number of young people with life-limiting conditions, but the numbers of young people aged 14–19 years with a life-limiting condition diagnosed in childhood showed a greater proportional increase in this study compared to the previous study. This may be due to the previous study not separating those with conditions first diagnosed in childhood from those with conditions first diagnosed in adulthood. If increases in survival, rather than incidence, are the main driver for increased numbers of young people with life-limiting conditions then it would be expected that there would be a greater increase in those with life-limiting conditions diagnosed in childhood (driven by increased survival) than those diagnosed as adults (driven mostly by incidence).

In contrast to the previous study looking at a 0–19 year old population with life-limiting conditions^1^, there was a close to even distribution across the deprivation categories in this study, rather than larger numbers in the most deprived categories. This may be due to differences in conditions (with differing life expectancies) between deprivation categories or due to differences in survival times for a given condition dependent on deprivation category. There is evidence of differential survival or progression for some conditions depending on deprivation status^32,33^ although not all studies show this^34^.

While there are no other studies looking nationally at medical complexity among young people with life-limiting conditions at transition ages in England, there is a study looking at complexity among children and young people with disabilities at a single centre^23^. This used a measure of complexity combining numbers of conditions, family reported issues and technology dependence. It found that the children most commonly had one to three issues, with a decrease in numbers with more issues but a significant group with 10 or more issues. This is similar to some of the findings in the present study, for example that young people of transition ages are most likely (18.0%) to have two distinct chronic conditions reported, a drop off in numbers with more conditions but a significant group (14.0%) with eight or more conditions. Also, while numbers of young people decreased with increasing numbers of consultant main specialities recorded in a year, 11.0% had more than six. These young people with greater medical complexity may be expected to have more healthcare use^23^.

There are also studies from North America that quantify numbers of children with medical complexity^21,35–37^. Unlike the present study, these studies have not looked at trends in complexity over time, but have attempted to quantify sizes of populations with medical complexity, under varying definitions, finding between 0.4% and 6% of study populations to be complex. The present study did not define a cut-off for having or not having medical complexity, focusing instead on whether there were changes in the level of complexity over time.

There was a lack of evidence for increases in inpatient healthcare use per person in the present study. This corresponds with previous findings on falls in length of stay and bed days per person in a related population with neurological conditions^38^ and increased clinical stability in young people with life-limiting conditions in Scotland^4^. Increased survival times for life-limiting conditions may be accompanied by increased time in a relatively stable state - while young people with greater medical complexity may increasingly be surviving to transition ages, those who were already surviving to these ages may be more stable than in the past. Better management in the community, in primary care and allied services, may reduce the need for inpatient admissions in this population^39^. Inpatient admissions and A&E visits are still much higher among young people with life-limiting conditions than in the general population^24,25,40^.

This study found increases in the numbers of young people with gastrostomies present, in line with previous analyses of HES data in England^41^ and similar data in a related population in Australia^42^. In line with the Australian study, there was little evidence for changes in the proportions of young people with life-limiting conditions at transition ages with gastrostomies. The increase in absolute numbers does however represent an increasing number of young people with high care needs. Estimated numbers with tracheostomies from the present study are comparable to that in a recent report on long term ventilation^43^, but that report looked at ages 0-24 years and was not restricted to those with life-limiting conditions. It did, however, only collect data from hospitals considered long term ventilation centres, in contrast to the present study using data from all NHS hospitals in England. The present study may also include some individuals not on long term ventilation.

Other studies have looked at overlap between indicators of complexity^23,24^. Although measures differed, they also found that multiple chronic conditions were associated with greater levels of technology dependence, including gastrostomies^24^ and that it is not unusual for a young person with complex healthcare needs to present with only one or two aspects of complexity^23^. Multiple measures are needed to identify young people with complex healthcare needs.

### Implications for policy

This study shows that the population with life-limiting conditions likely to need transition to adult care is increasing rapidly. This is particularly true for the non-White ethnic groups, underlying the importance of transition programmes that serve all sectors of the community. There were also differences in the rate of increase by Government Office Region, suggesting some areas may have greater increases than others in resource use for young people of these ages.

Increasing numbers of chronic conditions present and numbers of distinct consultant main specialties required for care has implications for the complexity of transition. A number of transitions between care teams treating different aspects of a young person’s condition and associated co-morbidities are likely to take place. This increases the need for coordination between care teams, not only paediatric and adult but also across different specialties, which may improve care and reduce unnecessary healthcare use^24,25,44–47^.

While there was a lack of evidence for increases in some aspects of hospital healthcare use per person, the rapidly increasing size of the population means that absolute numbers of admissions, emergency admissions, A&E visits and bed days are increasing greatly. This study also shows that one third of children and young people with life-limiting conditions are in the top 10% on at least one measure of complexity. Service planners will need to be aware of this.

There is evidence that emergency healthcare use increases when young people with life-limiting conditions in England transition to adult care^48^ and wider evidence for an increase in A&E visits^49^. As the population undergoing transition increases in size, this will increase pressures on healthcare systems and mean that a larger group is impacted by any negative experiences of emergency care. It is increasingly important for experiences of young people and for efficient healthcare resource to optimise transition processes.

### Implications for future research

This research leaves some unanswered questions. There is a disconnect between the apparent increasing complexity of the population reaching transition ages, at least in terms of diagnoses and numbers of consultants from different specialities involved in care and the lack of evidence for similar increases in hospital care use. Further research looking at individual conditions or closely related groups of conditions and at the last few years of life would be needed to assess whether increased survival is accompanied by longer periods of stability and whether this varies across conditions. In addition, there may be a lack of specificity in the coding framework for life-limiting conditions, due to a lack of specificity in ICD-10 diagnostic codes or due to changes in the diagnoses that should be considered life-limiting. Studies including primary care may give insights into whether care is moving more into the community for this population, with perhaps more or more frequent primary care contact replacing hospital admissions. Analysis of community prescribing would also provide information on whether patterns of prescribing are indicative of an increasingly stable population and potentially give insights on other aspects of complexity, e.g. polypharmacy^20^.

There is also a need for additional qualitative research in this population to understand medical complexity as experienced by young people, families, and clinicians and for research to develop better methods of measuring these from the available data. Smaller studies reviewing medical records or using mixed methods may provide greater insights, particularly on aspects of medical complexity not investigated in this study.

### Strengths and limitations

This study has a number of strengths. It draws upon established frameworks for defining medical complexity and identifying young people with life-limiting conditions. It uses national, whole-population data and so is representative with respect to the population of England. The methods are reproducible, enabling further updates to monitor changes into the future or application of alternative conceptualisations of medical complexity.

There are also limitations. Notably, several aspects of medical complexity previously identified^20^ cannot be assessed at all with the data used. In particular, the data are silent on family experiences and on measures of condition severity. As suggested above, these might be addressed through smaller and mixed method studies. There are also inevitable limitations to analysing data on a large scale, in the level of detail possible in defining life-limiting conditions, categories of life-limiting conditions and categories of distinct chronic conditions. While these were based on previously developed frameworks, other categorisations would be possible and may result in different findings.

## Conclusions

The group of young people with life-limiting conditions reaching ages at which transition to adult healthcare should take place is increasing rapidly, more quickly than for the population of children and young people with life-limiting conditions as a whole. This group is also increasing in medical complexity as far as numbers of conditions and numbers of consultant main specialties required for treatment and one third of included young people were in the top 10% for at least one measure of complexity. The increasing size of the group also means that use of hospital care, including emergency care, is increasing. There is limited overlap between measures of complexity, so multiple measures are required.

Healthcare providers will need to allocate resources to deal with increasing healthcare demands and greater complexity in conditions present and numbers of different care teams involved. Transition to adult healthcare must be managed well to limit impacts on healthcare resource use and improve experiences for young people and their families.

## Supplementary Material

Full Text XML

## Figures and Tables

**Figure 9:**
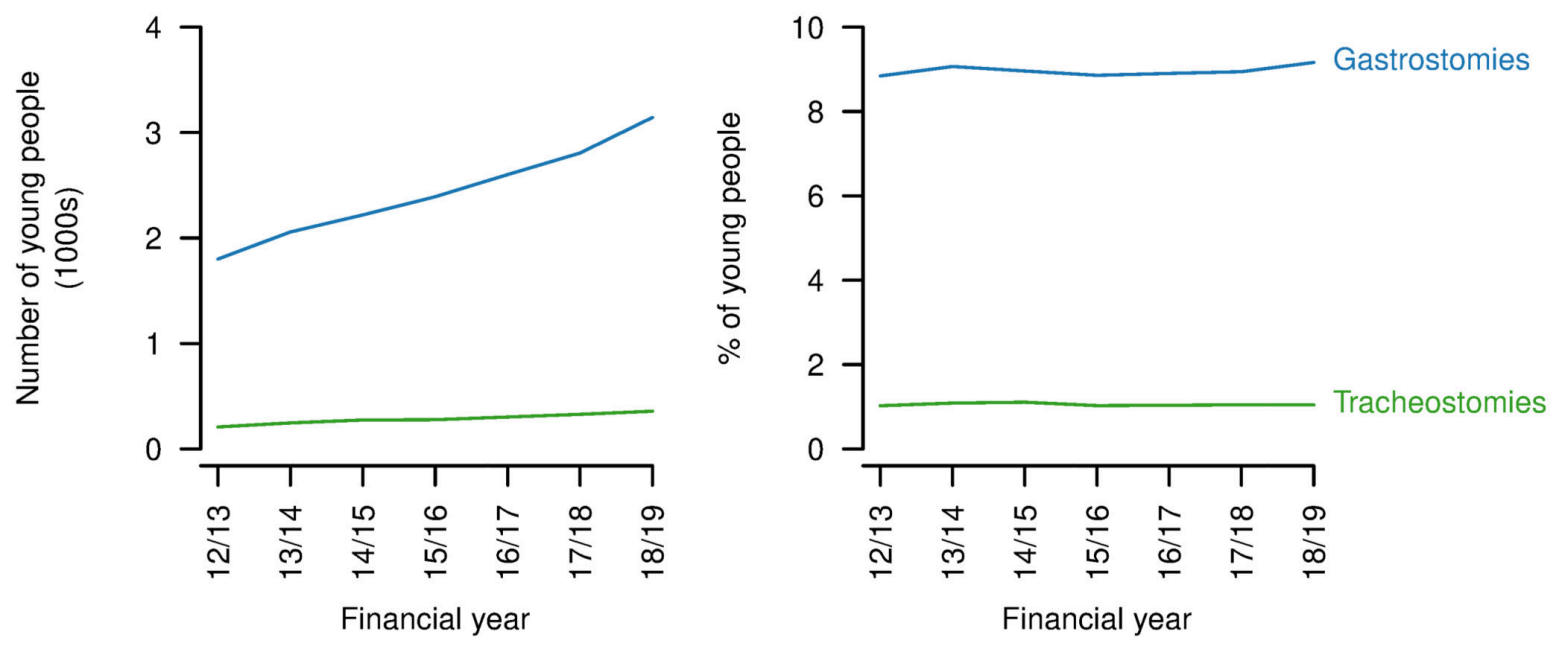
Technology dependence for young people aged 14–19 years with a life-limiting condition. Numbers (left) and proportions (right) of young people aged 14–9 years with a life-limiting condition also having a gastrostomy or tracheostomy present in each year.

**Table 1:** Coding framework for life-limiting conditions.

Diagnostic Group	ICD-10 diagnostic codes
**Neurology**	A17 A810 A811 F803 F842 G10 G111 G113 G12 G20 G230 G238 G318 G319 G35 G404 G405 G600 G601 G702 G709 G710 G711 G712 G713 G800 G808 G823 G824 G825 G934 G936 G937
**Haematology**	B20 B21 B22 B23 B24 D561 D610 D619 D70 D761 D81 D821 D83 D891
**Oncology**	C D444 D48 (Central Nervous System: C70, C71, C72, D33, D43)
**Metabolic**	E310 E348 E702 E71 E72 E74 E75 E76 E77 E791 E830 E880 E881
**Respiratory**	E84 J841 J96 J984
**Circulatory**	I21 I270 I42 I613 I81
**Gastrointestinal**	K550 K559 K72 K74 K765 K868
**Genitourinary**	N17 N184 N185 N19 N258
**Congenital**	Q000 Q01 Q031 Q039 Q040 Q042 Q043 Q044 Q046 Q049 Q070 Q200 Q203 Q204 Q206 Q208 Q213 Q232 Q218 Q220 Q221 Q224 Q225 Q226 Q230 Q234 Q239 Q254 Q256 Q262 Q264 Q268 Q282 Q321 Q336 Q396 Q410 Q419 Q437 Q442 Q445 Q447 Q601 Q606 Q614 Q619 Q642 Q743 Q748 Q750 Q772 Q773 Q774 Q780 Q785 Q792 Q793 Q804 Q81 Q821 Q824 Q858 Q860 Q870 Q871 Q872 Q878 Q91 Q920 Q921 Q924 Q927 Q928 Q932 Q933 Q934 Q935 Q938 Q952
**Other**	H111 H498 H355 M313 M321 M895 T860 T862 Z515

ICD-10 diagnostic codes were used to identify life-limiting conditions in this study. Where codes shorter than four digits are quoted, all four-digit sub-codes are included. ICD-10: International Classification of Diseases, 10th Edition.

**Table 2:** Grouping of chronic conditions to count numbers of distinct chronic conditions.

Category	ICD-10 codes
Substance abuse	E244, F10-F19, F55, G240, G312, G405, G621, G720, G721, I426, K292, K70, K852, K853, K860, O354, R781-R785, Y47, Y49, Z502, Z503, Z714, Z715, Z722, Z864
Self-harm	X60-X84, Y10-Y34, Y870, Y872, Z915
Other mental health problems	F00-F01, F028, F03-F09, F20-F48, F50, F53, F54, F59, F60-F69, F99, Z093, Z504, Z865, Z914
Behavioural/ developmental disorders	F70-F79, F800-F802, F808, F809, F81-F84, F88, F89, F90-F98
Neoplasms	C00-C97, D00-D02, D05-D09, D12, D13, D141-D144, D15, D20, D32-D35, D37-D48, D630, E340, E883, G130, G131, G533, G550, G631, G731, G732, G941, M360, M361, M495, M820, M906, M907, N081, N161, Y431-Y433, Y842, Z08, Z510-Z512, Z541, Z542, Z85, Z860, Z923
Immunological disorders	D80-D84, G532, Q980
Anaemia and other blood disorders	D50, D560-D562, D564, D568, D569, D570-D572, D578, D58, D610, D619, D64, D66, D67, D680-D682, D684-D689, D69, D70-D76, M362-M364, M904, N082, Z862
HIV	B20-B24, F024, R75, Z21
Other chronic infections	A50, A81, B18, B371, B375, B376, B377, B381, B391, B401, B440, B447, B45, B46, B487, B500, B508, B510, B518, B528, B520, B55, B572-B575, B580, B59, B67, B69, B73, B74, B787, B90-B94, F021, K231, K931, M00, N330, P350-P352, P358, P359, P37
Asthma and chronic lower respiratory disease	J41-J47
Cystic flbrosis	E84, P75
Respiratory injuries	S17, S27, S28, T27, T914
Respiratory congenital anomalies	Q30-Q37, Q790
Other respiratory	G473, J60-J70, J80-J86, J961, J98, P27, Y556, Z430, Z930, Z942
Diabetes	E10-E14, G590, G632, I792, M142, N083, O24, Y423
Other endocrine	E00, E030, E031, E071, E220, E230, E25, E268, E291, E31, E341, E342, E345, E348, G132, G735, Y421
Metabolic	D55, E70-E72, E74-E78, E791-E799, E800-E803, E805, E807, E83, E85, E880, E881, E882, E888, E889, G736, L990, M144, M143, N163
Digestive	K20, K210, K22, K238, K25-K28, K290, K291, K293-K299, K31, K50-K52, K55, K57, K592, K630-K633, K66, K72-K76, K80-K83, K850, K851, K858, K859, K861-K869, K870, K90, M074, M075, M091, M092, T864, Z432-Z434, Z465, Z903, Z904, Z932-Z935
Renal/ genitourinary	D638, G638, G998, I688, M908, N084, N00-N05, N07, N11-N15, N160, N162, N164, N165, N168, N18, N19, N20-N23, N25, N26, N28, N29, N31, N32, N338, N35, N36, N391, N393, N394, N40-N42, N70-N74, N80-N82, N85, N86, N87, N88, P960, T824, T831, T832, T834-T839, T855, T861, Y602, Y612, Y622, Y841, Z49, Z936, Z940, Z992
Congenital anomalies of the digestive/ renal/ genitourinary system	Q380, Q383, Q384, Q386-Q388, Q39, Q402, Q403, Q408, Q409, Q41, Q42, Q431, Q433-Q437, Q439, Q44, Q45, Q500, Q51, Q520-Q522, Q524, Q540-Q543, Q548, Q549, Q550, Q555, Q56, Q601, Q602, Q604-Q606, Q61, Q620-Q626, Q628, Q630-Q632, Q638, Q639, Q64, Q792-Q795, Q878, Q891, Q892
Digestive/ renal/ genitourinary injuries	S36, S37, S38, S396, S397, T065, T28, T915
Other/ unspecific metabolic/ endocrine/ digestive/ renal/ genitourinary	E66, G633, G990, M145, N92, Z863, Z938
Musculoskeletal/ connective tissue	G551-G553, G635, G636, G737, J990, J991, L620, M05, M06, M070-M073, M076, M08, M098, M10-M13, M140, M146, M148, M30-M35, M40-M43, M45-M48, M50-M54, M60-M62, M638, M801-M809, M811-M819, M821, M828, M840-M842, M848, M849, M85, M863-M866, M89, M900, M91-M94, N085, Y454
Skeletal injuries/amputations	S13, S220-S222, S225, S23, S32, S33, S683, S684, S688, S77, S78, S87, S88, S97, S980, S982-S984, T02, T04, T05, T203, T207, T213, T217, T223, T227, T232, T233, T236, T237, T243, T247, T252, T253, T256, T257, T293, T297, T303, T307, T312-T319, T322-T329, T873-T876, T912 T918, T926, T931, T934, T936, T940, T941, T950, T951, T954, T958, T959, Y835, Z891, Z892, Z895-Z898, Z971
Chronic skin disorders	L10, L110, L118, L119, L12-L14, L28, L40-L45, L57, L581, L59, L87, L88, L90, L92, L95, L93, L985, M090, Q80, Q81, Q870-Q875, Q894
Musculoskeletal/ skin congenital anomalies	Q188, Q650-Q652, Q658, Q659, Q675, Q682, Q683-Q685, Q71-Q73, Q74, Q753-Q759, Q761-Q764, Q77, Q78, Q796, Q798, Q820-Q824, Q829, Q862, Q897-Q899
Epilepsy	F803, G400-G404, G406-G409, G41, R568, Y460-Y466
Cerebral palsy	G80-G83
Injuries of brain, nerves, eyes or ears	S05-S08, S12, S14, S24, S34, S44, S54, S64, S74, S84, S94, T060-T062, T26, T904, T905, T911, T913, T924
Chronic eye conditions	H051-H059, H133, H17, H18, H193, H198, H21, H26, H27, H280-H282, H31, H328, H33, H34, H35, H40, H420, H43, H44, H47, H540- H542, H544, T852, T853, Z442
Chronic ear conditions	H602, H652-H654, H661-H663, H690, H701, H731, H740-H743, H750, H80, H810, H814, H830, H832, H900, H903, H905, H906, H91, Z453
Congenital anomalies of neurological or sensory systems	Q00-Q07, Q104, Q107, Q11-Q12, Q130-Q134, Q138, Q139, Q14-Q16, Q750, Q751, Q85, Q860, Q861, Q868, Q90-Q93, Q952, Q953, Q97, Q99
Other neurological	F022, F023,G00-G09, G10-G12, G138, G14, G20-G23, G241-G249, G25-G30, G310-G311, G318, G319, G32-G37, G43-G46, G470-G472, G474-G479, G50-G52, G530, G531, G538, G54, G558, G56-G58, G598, G60, G61, G620, G622-G629, G64, G70, G71,G722-G729, G730, G733, G90-G93, G942, G948, G95, G96, G98, G991, G992, I60-I67, I680, I682, I69, I720, I725, T850, T851, Y467-Y468, Z982
Congenital heart disease	Q20-Q26, Q893
Other cardiovascular	I00-I28, I31-I39, I41, I420-I425, I427-I429, I430, I431, I432-I438, I441-I447, I451-I459, I46-I51, I528, I70-I71, I721-I724, I728, I729, I73-I77, I790, I791, I798, I81-I82, I98-I99, M036, N088, Q27, Q28, S26, T820-T823, T825-T829, T862, Y605, Y615, Y625, Y840, Z450, Z500, Z941, Z95
Non-specific chronic conditions	R62, R633, Z431, Z515, Z755, Z931, Z993

ICD-10 diagnostic codes for chronic conditions (including life-limiting conditions) grouped into categories for the purpose of counting numbers of distinct chronic conditions. ICD-10: International Classification of Diseases, 10th Edition; HIV: human immunodeficiency virus.

**Table 3:** Related paediatric and adult consultant main specialties.

Codes	Descriptions of specialties
141, 142, 149	Restorative Dentistry; Paediatric Dentistry; Surgical Dentistry
320, 321	Cardiology; Paediatric Cardiology
400, 421	Neurology; Paediatric Neurology
710, 711	Adult Mental Illness; Child and Adolescent Psychiatry

Consultant main specialties spanning paediatric and adult disciplines that were treated as a single specialty for the purposes of counting distinct consultant main specialties each year. Codes are from the Hospital Episode Statistics Technical Output Specification (https://digital.nhs.uk/data-and-information/data-tools-and-services/data-services/hospital-episode-statistics/hospital-episode-statistics-data-dictionary) for field ‘MAINSPEF’ in Admitted Patient Care and Outpatient datasets

**Table 4:** Codes used to identify presence of gastrostomies and tracheostomies.

Coding system	Code	Interpretation
ICD-10	Z430	Tracheostomy present and assumed to remain until evidence of removal
ICD-10	Z930	Tracheostomy present and assumed to remain until evidence of removal
OPCS-4	E421	Tracheostomy present and assumed to remain until evidence of removal
OPCS-4	E423	Temporary tracheostomy - counted as present in year, but not in subsequent years unless evidence of subsequent permanent tracheostomy
OPCS-4	E425	Tracheostomy removed - counted as present in year, but not in subsequent years unless evidence of subsequent reinsertion
OPCS-4	E426	Tracheostomy present and assumed to remain until evidence of removal
OPCS-4	E427	Tracheostomy removed - counted as present in year, but not in subsequent years unless evidence of subsequent reinsertion
ICD-10	Z431	Gastrostomy present and assumed to remain until evidence of removal
ICD-10	Z931	Gastrostomy present and assumed to remain until evidence of removal
OPCS-4	G341	Gastrostomy present and assumed to remain until evidence of removal
OPCS-4	G342	Temporary gastrostomy - counted as present in year, but not in subsequent years unless evidence of subsequent permanent gastrostomy
OPCS-4	G343	Gastrostomy present and assumed to remain until evidence of removal
OPCS-4	G344	Gastrostomy removed - counted as present in year, but not in subsequent years unless evidence of subsequent reinsertion
OPCS-4	G345	Gastrostomy present and assumed to remain until evidence of removal
OPCS-4	G445	Gastrostomy present and assumed to remain until evidence of removal
OPCS-4	G447	Gastrostomy removed - counted as present in year, but not in subsequent years unless evidence of subsequent reinsertion

ICD-10 and OPCS-4 codes considered indicative of presence of gastrostomies or tracheostomies in a year and/or following years. ICD-10: International Classification of Diseases, 10th Edition; OPCS-4: OPCS Classification of Interventions and Procedures Version 4.

## Data Availability

The data used in this study are personal healthcare data used under agreement from NHS Digital. The individual data cannot be shared under the data sharing agreement. Other researchers are free to apply for similar data from NHS Digital through the Data Access Request Service. The data requested from NHS Digital were all records up to age 23 years for all children and young people who were aged 12–23 years at any point between 1 April 2007 – 31 March 2019 from the following datasets: Hospital Episode Statistics ‘Admitted Patient Care’ dataset (records from 1 April 2006 – 31 March 2019) Hospital Episode Statistics ‘Outpatient’ dataset (records from 1 April 2006 – 31 March 2019) Hospital Episode Statistics ‘Accident & Emergency’ Department dataset (records from 1 April 2007 – 31 March 2019

## Plain english summary

Life-limiting conditions are conditions that shorten or threaten to shorten life. Children with these conditions receive healthcare from specialist children’s services. As adults, they get treated in adult services, often overseen by a General Practitioner (GP). The transition often happens between 14 and 19 years.

Healthcare providers need information on these young people to provide good services. We don’t know much about how many there are, what conditions they have, whether they are male or female, what ethnic group they are in or where they live. We also don’t know whether their healthcare needs are becoming more complex.

We aimed to discover how many of the children survive into adulthood. We also wanted to know the number of people from different ethnic groups, regions, areas of high or low deprivation and how many were male and female. We looked at how complicated healthcare needs were by counting how many long-term conditions they had and how many different care teams were involved. We also counted admissions to hospital and visits to Accident & Emergency (A&E) Departments and how many needed technology to help with eating or breathing. This was all done using records routinely collected by the NHS.

The number of young people with life-limiting conditions surviving to adulthood had increased. There were 20363 in 2012/13 and 34307 in 2018/19. The number from minority ethnic groups had increased, particularly the Mixed and Pakistani groups. The young people had more long-term conditions as time went on. They also had more different medical teams involved in care. They had more visits to A&E Departments. Admissions to hospitals per person had not increased.

Healthcare providers need to be aware of these changes. Increasing numbers make it more important to get transition right. Increasing numbers of conditions and medical teams involved make this more difficult.
