# Supplementary material for: Numbers, characteristics, and medical complexity of children with life-limiting conditions reaching age of transition to adult care in England: a repeated cross-sectional study
Source: NIHR Open Res. 2022 Apr 8;2:27. [Version 1] doi: 10.3310/nihropenres.13265.1 (PMC7613215; doi:10.3310/nihropenres.13265.1)
Supplement: Peer review history

# Reviewer report: Reviewer response for version 1

**DOI:** 10.3310/nihropenres.14384.r28696

**Published:** 2022-07-18

**Authors:** Adelaide Withers

**Affiliations:** 1 Department of Respiratory and Sleep Medicine, Perth Children’s Hospital, Perth, WA, Australia; 2 Wal-yan Respiratory Research Centre, Nedlands, WA, Australia; 3 Curtin University, Perth, WA, Australia

This study examines young people with a life limiting illness between 2012-2019. The comparison between these time periods examines number of young people and the complexity of their medical conditions. Assessment of complexity considered the total number of medical conditions, number of specialities involved, hospital admissions, emergency visits, LOS, technology dependence. The authors found an increase in the number of young people with life limiting illnesses during this time and increased complexity from co morbid conditions and number of specialities involved. This has implications for transition planning and resource allocation. They attribute the increase in numbers to more young people with life limiting conditions surviving to the age where transition would occur. This is consistent with the observed increase in complexity.

 This was a generally well written manuscript that was novel and relevant. I did find the presentation of results regarding the top 10% of measures of complexity on page 10 and 12 a little confusing, particularly the last few sentences as there are so many percentages in each sentence. I had to read this section several times to fully understand it.

 I really liked the diagram in Figure 2 representing the layers of medical complexity, this is fantastic.

 I did feel as if some tables would be better in an appendix, and others may be clearer if some examples were given. In Table 1, listing the ICD-10 codes for life limiting conditions, I actually looked a lot of these codes up as I wasn't sure which conditions would have counted for neurology (as an example). I feel it may be easier for the reader to have a table with a list of major diagnoses for each group and include IDC-10 codes in an appendix. Table 2 could go in an appendix, or even just keep the list of categories rather than include ICD-10 codes. Similarly, Table 4 could go in an appendix. I found these tables distracting. 

 On page 4, I wasn't sure why some dates were 1 April 2006 and other were 1 April 2007. On page 10 under function limitations there appears to be a typographical error as twice it says 2012/23 instead of 2012/13. 

 The discussion is clear and considered. It would be interesting to include speculation as to why the numbers increased in certain ethnic groups and decreased in others. I thought it was interesting that there wasn't an increased proportion with tracheostomies. This could be due to increased use of non-invasive ventilation/home ventilation in conditions that previously would have required tracheostomies. 

 The strengths and limitations are well discussed. The conclusions are consistent with the data and the discussion.

 This is well written and informative manuscript that highlights some of the challenges faced during the transition period, and adds valuable data will assist in service and resource allocation for these increasingly complex young adults.

Is the work clearly and accurately presented and does it cite the current literature?

Yes

If applicable, is the statistical analysis and its interpretation appropriate?

Yes

Are all the source data underlying the results available to ensure full reproducibility?

Yes

Is the study design appropriate and is the work technically sound?

Yes

Are the conclusions drawn adequately supported by the results?

Yes

Are sufficient details of methods and analysis provided to allow replication by others?

Yes

Reviewer Expertise:

Paediatric respiratory and sleep, neuromuscular disorders, respiratory failure, non invasive and invasive ventilation, adolescent patients

I confirm that I have read this submission and believe that I have an appropriate level of expertise to confirm that it is of an acceptable scientific standard.

# Reviewer report: Reviewer response for version 1

**DOI:** 10.3310/nihropenres.14384.r28614

**Published:** 2022-05-24

**Authors:** Intira Jeannie Collins

**Affiliations:** 1 Medical Research Council Clinical Trials Unit, Institute of Clinical Trials and Methodology, University College London, London, UK

This paper by Jarvis *et al.* is based on analyses of NHS digital data including Hospital Episode Statistics on ‘Admitted Patient Care’, ‘Outpatient’ care and Hospital Episode Statistics on Accident & Emergency in England. The paper describes trends over calendar years (2012-2019) in the number and characteristics young people aged 14-19 years with life-limiting conditions, who were in the age group preparing to transition to adult care. The paper provides a detailed descriptive summary of the disease areas, characteristics (sex, ethnicity, geographic region, etc) and how this has evolved over time. The authors also explored different indicators of medical complexity based on number of conditions/co-morbidities, number of specialities of consultants involved in their care and the number of hospital admissions/A&E visits per person, with the aim of informing future care needs. 

 The paper is well written, the analyses are sound and the results were presented in a clear and novel manner. The strengths and limitations were well described. 

 One issue is that the term 'life-limiting condition' is very broad, more detail of the specific disease areas dominating key areas or specific disease which has seen marked changes in the burden over time would be even more informative e.g. decline in HIV diagnosis in childhood. It would also be informative to understand if some of the burden of hospital admissions/A&E visits are disproportionately due to key disease areas or conversely if some disease areas observed large declines in need of complex care - this would be highly informative for future care planning. Lastly, predictors of populations at risk of frequent hospital admissions and extended admissions would be particularly informative but the authors did acknowledge that as a limitation and an area for future research.

Is the work clearly and accurately presented and does it cite the current literature?

Yes

If applicable, is the statistical analysis and its interpretation appropriate?

Yes

Are all the source data underlying the results available to ensure full reproducibility?

Yes

Is the study design appropriate and is the work technically sound?

Yes

Are the conclusions drawn adequately supported by the results?

Yes

Are sufficient details of methods and analysis provided to allow replication by others?

Yes

Reviewer Expertise:

Paediatric infectious diseases, epidemiology

I confirm that I have read this submission and believe that I have an appropriate level of expertise to confirm that it is of an acceptable scientific standard.

# Response

**Published:** 2022-05-26

**Authors:** Stuart Jarvis

**Affiliations:** University of York, UK, UK

We thank the reviewer for these kind and helpful comments.

 As noted, predictors of medical resource use are beyond the scope of this paper, but certainly relevant ot future research. Previous cited publications go into a little more detail on the 'life-limiting conditions' included and how they were defined. We do however agree that it would be useful to see, for example, how the mix of most prevalent conditions has changed over time and perhaps the medical resource burden associated with these. We will look at including this information in a revised version once all peer review reports are in.
